# Efficacy of Interceptor^®^ G2, a new long-lasting insecticidal net against wild pyrethroid-resistant *Anopheles gambiae s.s.* from Côte d’Ivoire: a semi-field trial

**DOI:** 10.1051/parasite/2018042

**Published:** 2018-08-08

**Authors:** Soromane Camara, Ludovic Phamien Ahoua Alou, Alphonsine Amanan Koffi, Yao Cyntia Muriel Clegban, Jean-Paul Kabran, Fernand Mathieu Koffi, Kouakou Koffi, Cédric Pennetier

**Affiliations:** 1 Institut Pierre Richet/Institut National de Santé Publique (INSP) Bouaké Côte d’Ivoire; 2 Université Félix Houphouët-Boigny Abidjan Côte d’Ivoire; 3 Institut de Recherche pour le Développement (IRD), Maladies Infectieuses et Vecteurs, Ecologie, Génétique, Evolution et Contrôle (MIVEGEC), UMR 224 Bouaké Côte d’Ivoire

**Keywords:** Malaria, *Anopheles gambiae s.s.*, insecticides, chlorfenapyr, pyrethroid resistance

## Abstract

*Background*: The widespread insecticide resistance in malaria vector populations is a serious threat to the efficacy of vector control tools. As a result, the World Health Organization (WHO) supports the development of alternative tools that combine several insecticides with the aim of improving vector control and the management of insecticide resistance. In the present study, a long-lasting insecticidal net treated with a mixture of chlorfenapyr and alphacypermethrin was evaluated against wild pyrethroid-resistant *Anopheles gambiae s.s* in M’bé, Côte d’Ivoire. Centers for Disease Control and Prevention (CDC) bottle tests were carried out with resistant *An. gambiae s.s.* of M’bé and the susceptible strain, to assess the resistance level to chlorfenapyr and alphacypermethrin. *Results*: CDC bottle bioassays revealed a high level of resistance of *An. gambiae s.s.* population from M’bé to alphacypermethrin, whereas they revealed low resistance to chlorfenapyr. In experimental huts, Interceptor^®^ G2 that was unwashed or washed 20 times killed 87% and 82% of *An. gambiae s.s.*, respectively, whereas Interceptor^®^ LN that was either unwashed or washed 20 times killed only about 10% of the mosquitoes. The blood-feeding inhibition induced by Interceptor^®^ was not significantly different compared to untreated nets, whereas Interceptor^®^ G2 that was unwashed or washed 20 times induced 42% and 34% inhibition of blood-feeding, respectively. *Conclusion*: Interceptor^®^ G2 met the WHOPES criteria to undergo a phase III study. Investigation of its efficacy at a community level and the conduct of randomized controlled trials dealing with epidemiological outputs are warranted in order to study the potential of Interceptor^®^ G2 to better protect communities.

## Abstract (fr)

*Introduction* : La résistance aux insecticides, répandue dans les populations de vecteurs du paludisme, est considérée comme une grave menace pour l’efficacité des outils de lutte antivectorielle. Par conséquent, l’Organisation Mondiale de la Santé encourage le développement de nouveaux outils avec des combinaisons insecticides pour améliorer la lutte antivectorielle et gérer la résistance aux insecticides. Dans la présente étude, une moustiquaire à longue durée d’action (MILDA), imprégnée avec un mélange de chlorfénapyr et alpha-cyperméthrine a été évaluée contre la population naturelle d’*Anopheles gambiae* de M’bé, Côte d’Ivoire. Des tests en bouteilles CDC ont été réalisés avec la population résistante d’*An. gambiae s.s.* de M’bé et la souche sensible pour déterminer le niveau de résistance au chlorfénapyr et à l’alpha-cyperméthrine. *Résultats* : tests en bouteilles CDC ont montré un niveau élevé de résistance de la population naturelle d’*An. gambiae* de M’bé à l’alphacyperméthrine mais plus faible au chlorfénapyr. En cases expérimentales, Interceptor^®^ G2 non lavée et lavée 20 fois a tué respectivement 87 % et 82 % d’*An. gambiae s.s.* tandis qu’Interceptor^®^ non lavée et lavée 20 fois n’a tué seulement qu’environ 10 % des moustiques. L’inhibition du taux de gorgement induite par Interceptor^®^ n’a pas été significativement différente de celle de la moustiquaire non traité alors qu’Interceptor^®^ G2 non lavée et lavée 20 fois a induit respectivement 42 % et 34 % d’inhibition de gorgement. *Conclusion* : Interceptor^®^ G2 répond aux critères du WHOPES pour une étude en phase III. L’étude de son efficacité au niveau communautaire et des essais randomisés contrôlés traitant des données épidémiologiques sont nécessaires afin d’étudier la capacité d’Interceptor^®^ G2 à mieux protéger les communautés.

## Introduction

Malaria remains a serious public health burden in endemic regions. The World Health Organization (WHO) estimates that there were 214 million new cases of the disease and more than 445,000 malaria-related deaths in 2016, with 91% of these occurring in Africa [45]. As there are no commercially available vaccines against this disease, vector control remains crucial to reduce disease transmission. The Global Fund and the President’s Malaria Initiative (PMI) are supporting the scaling-up of long-lasting insecticidal nets (LLINs) and indoor residual spraying (IRS) in many endemic African countries [5, 8]. Long-lasting insecticidal nets (LLINs) have contributed significantly to the success of malaria control in such malaria-endemic countries. Since 2000, malaria mortality rates have fallen by 66% among all age groups and by 71% among children under 5 years of age [44]. According to WHO estimates, the incidence of malaria (i.e. the rate of new malaria cases) fell by 18% between 2010 and 2016 [45].

There are five main classes of neurotoxic insecticides that are recommended or prequalified by WHO for IRS (carbamates, neonicotinoids, organochlorines, organophosphates and pyrethroids), whereas only pyrethroids are recommended for insecticide-treated nets (ITNs) in light of their relatively low toxicity to humans, a rapid knockdown effect on mosquitoes, and low cost [41]. Since the introduction of pyrethroids for ITN impregnation in the 1980s, no new adulticide has been approved for ITN treatment [29].

Unfortunately, in the last decade pyrethroid resistance has become widespread in *Anopheles* genera in Sub-Saharan Africa [38]. This was mainly driven by the high selective pressure stemming from the massive use of pyrethroids in agriculture [11, 19] and the scaling-up of pyrethroid ITNs and IRS for malaria control [10, 39, 47]. Although the epidemiological impact of resistance mechanisms on vector control remains controversial, several reports of pyrethroid resistance have revealed reduced vector mortality and a consequent drastic loss of personal protection conferred by pyrethroid-treated nets to humans [2, 25]. From 2010 to 2016, 61 countries reported mosquito resistance to at least one insecticide used in nets and indoor residual spraying [45]. This situation represents a serious threat to the efficacy of malaria control tools. The WHO recommends the use of insecticide combinations with different modes of action for LLIN impregnation in order to manage pyrethroid resistance in malaria vectors; the underlying hypothesis is that insects that can survive contact with one component of the mixture would be killed by the second insecticide [12, 18, 36, 42].

In this context, two concomitant objectives are to manage resistance and to maintain the protective efficacy of LLINs. Alternative insecticides with novel modes of action are available for which there are no reports of resistance in malaria vectors. Chlorfenapyr, a pyrrole insecticide, is one of these. It is used commercially for termite control and crop protection against a variety of insect and mite pests [40]. It exerts its action by targeting the oxidative pathways in the insect’s mitochondria, thereby disrupting ATP production [6]. As no cross-resistance between chlorfenapyr and existing classes of public health insecticides has been reported to date, its novel mode of action makes it a suitable candidate insecticide for targeting resistant malaria vectors that are multi-resistant [26, 37]. Chlorfenapyr has been shown to have the potential to provide improved control of pyrethroid-resistant *An*. *gambiae* both in laboratory and in controlled conditions against natural free-flying resistant malaria vectors [32]. A mixture of chlorfenapyr and alpha-cypermethrin on bed nets has been shown to provide excito-repellency and strong insecticidal activity against pyrethroid resistant mosquitoes [27, 31]. Recently, BASF^©^ developed a long-lasting insecticidal mixture net, called Interceptor^®^ G2, that is made of polyester fibers and treated with a mixture of alphacypermethrin/chlorfenapyr. The World Health Organization Pesticide Evaluation Scheme (WHOPES) reviews and makes recommendations on new pesticide technologies for public health programs, such as LLINs. The WHOPES testing and evaluation process is divided into four phases: Phase I in laboratory conditions, Phase II on wild vector populations in experimental field huts, Phase III is a three-year review of overall performance in the field, and Phase IV for development of WHO specifications for quality control for international trade. The new LLIN met the WHO efficacy criteria in laboratory tests against the susceptible Kisumu strain and pyrethroid-resistant *An. gambiae* strain [28]. This paper reports the results of an experimental hut trial in Côte d’Ivoire of Interceptor^®^ G2 against resistant *Anopheles gambiae s.s.* in terms of deterrence, induced exophily, blood-feeding inhibition, and mortality.

## Materials and methods

### Ethics clearance

Ethics approval was obtained from the Ministry of Health and Public Hygiene in Côte d’Ivoire through the National Research Ethics Committee (No. 052/MSHP/CNER-kp). Adult volunteers were recruited among the inhabitants of the villages close to the study site. After obtaining written informed consent, they were vaccinated against yellow fever. Medical supervision was provided during the trial and one month after the experimental hut trial by a qualified medical doctor. Confirmed malaria cases were treated according to national policies.

### Study site and the design of the huts

The study was conducted in experimental huts located in the M’Bé area (Côte d’Ivoire). The station, built by the Institute Pierre Richet to run WHOPES phase II studies, is located 30 km north of Bouaké (5.209963° W and 7.970241° N) in central Côte d’Ivoire. The Bouaké area is characterized by wet savannah with a single annual rainy season (April to October), an average annual rainfall of 1,200 mm, and an average temperature of 25.8 °C. The mosquito population in the area is composed of *An. coluzzii, An. gambiae*, *An. funestus*, *Culex sp*., and *Mansonia sp.* [17]. The *Anopheles gambiae s.s.* population is resistant to pyrethroids, organochlorides, and carbamates, with an allelic frequency of the L104F *kdr* mutation of around 80% and the presence of metabolic resistance mechanisms [7, 17].

The huts, which are typical of the West African region, are made of concrete blocks with a corrugated iron roof. They have a ceiling made of thick polyethylene sheeting, and they have a concrete foundation slab surrounded by a water-filled channel that is meant to prevent ants from entering the structure [15]. Mosquitoes, however, can readily enter through four window slits. These are made from metal that is fixed at an angle to create a funnel with a 1 cm-wide gap. During each evaluation, the window slits are opened from 8:00 pm to 5:00 am by the custodian. Mosquitoes must fly upward through the funnel to enter through the gap in the wall and into the hut, and downwards to exit, thereby impeding the exit of the majority of mosquitoes that entered the hut. A single veranda trap projecting from the back wall of each hut is also part of the design and it is made of polyethylene sheeting and screening mesh that is 2 m long, 1.5 m wide, and 1.5 m high. During the night, the mosquitoes can move unimpeded between the hut and veranda.

### Net treatments and experimental design

Interceptor^®^ G2 were factory coated with 100 mg/m^2^ alpha-cypermethrin and 200 mg/m^2^ chlorfenapyr, whereas Interceptor^®^ were factory coated with 200 mg/m^2^ alpha-cypermethrin. Six untreated polyester nets were used as a negative control and six others were hand-treated (CTN) with chlorfenapyr (200 mg AI/m^2^), using adequate protective equipment (i.e. gloves, a face mask, and goggles). The chlorfenapyr formulation (Phantom 240 g/L SC) was supplied by BASF SE (Germany). All of the nets were 100 Denier polyester.

The following six treatment arms were tested: 1) unwashed Interceptor^®^ G2; 2) Interceptor^®^ G2 washed 20 times; 3) unwashed Interceptor^®^; 4) Interceptor^®^ washed 20 times; 5) polyester nets hand-treated with chlorfenapyr at 200 mg/m^2^; and 6) untreated polyester nets. The nets were washed 20 times according to a protocol from the standard WHO washing procedure used in the phase II trial [43]. The interval of time required between two washes (i.e. the regeneration time) was one day, as established in phase I of the trial [46]. The nets were deliberately holed to simulate wear and tear. Six holes (4 cm × 4 cm) were made in each net, two holes in each of the long side panels, and one hole at each end (i.e. in the head- and foot-side panels). Six nets (one per night of the week) were used for each treatment arm.

Adult volunteers entered the hut at dusk and slept under the nets until dawn six nights per week.

The treatment arms were rotated among the huts each week and sleepers rotated each night according to a randomized Greco-Latin square scheme to minimize variations due to the hut and/or human attractiveness. At the end of the week, the huts were carefully cleaned and aired to avoid potential contamination. Each morning, resting and dead mosquitoes were collected from the inside of the nets, the room, and the veranda trap. The mosquitoes were morphologically identified to the species level using taxonomic keys. The malaria vectors were scored by location (room, veranda and mosquito net) as dead or alive and as fed or unfed. Live mosquitoes were placed in small cups and provided with access to a sugar solution for 72 h holding periods in order to assess delayed mortality every 24 h up to 72 h [24].

The following outcomes were measured to assess the efficacy of the treatments in the experimental huts:

- Deterrence (i.e. the reduction in the number of mosquitoes collected in the huts with treated nets relative to the control huts);
- Induced exophily (i.e. the reduction in the proportion of mosquitoes collected in the veranda traps relative to the control huts);
- Blood-feeding inhibition (i.e. the reduction in the proportion of blood-fed mosquitoes in the huts with treated nets relative to the control huts);
- Immediate and delayed mortality (i.e. the proportion of dead mosquitoes when collected in the morning and at 24–72 h after collection);
- Personal protection (i.e. the reduction in the number of blood-fed malaria vectors collected in the treated arms relative to the negative control), calculated as follows:

$$\%\;\mathrm{personal}\;\mathrm{protection}=\;100\times \;(B_{u}\;-B_{t}/\;B_{u})$$

where *B*_u_ is the total number blood-fed mosquitoes in the hut with untreated nets, and *B*_t_ is the total number blood-fed mosquitoes in the hut with treated nets.

### Reporting of adverse events

Volunteers were asked to report any adverse events associated with use of the treated nets, and they had access to medical care free of charge, if necessary.

### Insecticide resistance test

Centers for Disease Control and Prevention (CDC) bottle bioassays were performed to generate dose response data. Glass 250 mL bottles were coated with various concentrations of alpha-cypermethrin and chlorfenapyr according to CDC guidelines [24] to determine the level of resistance of the M’Bé population. The tests were carried out on both M’Bé and Kisumu adult mosquitoes. They were conducted at 27 °C ± 0.5 °C and 75% ± 10% relative humidity with batches of 25 *An. gambiae* females that were 3–5 days old. The mosquitoes were exposed for 1 h and mortality was recorded 24 h later and every 24 h up to 72 h later for alpha-cypermethrin and chlorfenapyr, respectively.

### LLINs bio-assays

Standard WHO cone bioassays were used to determine the bio-efficacy of LLINs against the susceptible Kisumu and the resistant M’Bé strain. Two nets per treatment were bio-assayed 1) before any washing, 2) after washes, and then 3) at the end of the field trial. Ten cones were placed on the five sections of each net (two per section). Five unfed 2–3-day old female mosquitoes were exposed for 3 min and 30 min in each cone; 3 minutes is the standard WHO specified exposure time for pyrethroid nets, whereas a prolonged exposure of 30 min may be more suitable for chlorfenapyr insecticide. Knockdown (KD) was checked 60 min after exposure and mortality was recorded every 24 h up to 72 h after exposure.

### Statistical analysis and data safety

The mortality data from the CDC bottle assays were analyzed using R Software with the script developed by Milesi and Labbé [21]. After testing the linearity of the dose-mortality responses and computation of its slope and standard deviation, the doses of insecticide required to kill 50% of the exposed mosquitoes (Lethal Concentration 50, or LC_50_) and the associated confidence intervals were calculated. Finally, this allowed for comparison of two dose-mortality lines and the calculation of resistance ratios, or RRs (= LC_50_ of the field sample/LC_50_ of the reference strain) and their 95% confidence intervals.

The mortality and the KD rates from the WHO cone bioassays were compared between each net using the *χ* ^2^ test. For statistical testing, the level of significance was set at 5%.

The proportional data (i.e. the induced exophily, blood-feeding inhibition, and induced mortality) of each treatment were analyzed with logistic regression models using the “brglm” function from the brglm package for R (version 3.3.2) on the basis of the bias-reduction method developed by Firth [13]. These procedures yield estimates with improved frequentist properties (e.g. bias and the mean squared error) that are always finite even in cases where the maximum likelihood estimates are infinite (data separation). The number of collected mosquitoes entering the huts (deterrence) and the number of blood-fed mosquitoes (personal protection) were analyzed using negative binomial regression with adjustment for the sources of variation between the huts, sleepers, the weeks of the trial, and for variation not explained by the other terms.

The field efficacy of Interceptor^®^ G2 was compared to untreated control nets and a commercial standard Interceptor^®^ approved by WHOPES. A chlorfenapyr hand-treated net was also used for comparison.

## Results

### Resistance status

**Figure 1.**
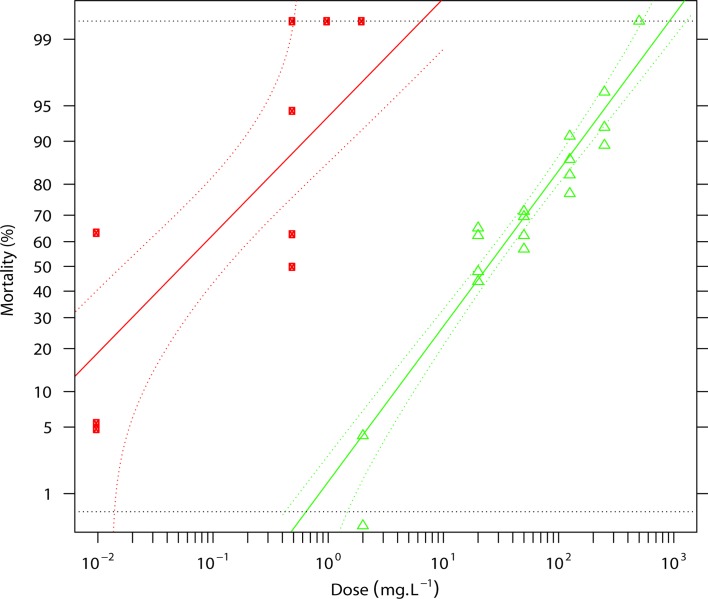
Regression line showing the mortality rates of the Kisumu and M’Bé strains to relative doses of alphacypermethrin. Red squares: Kisumu; green triangles: M’Bé.

**Figure 2.**
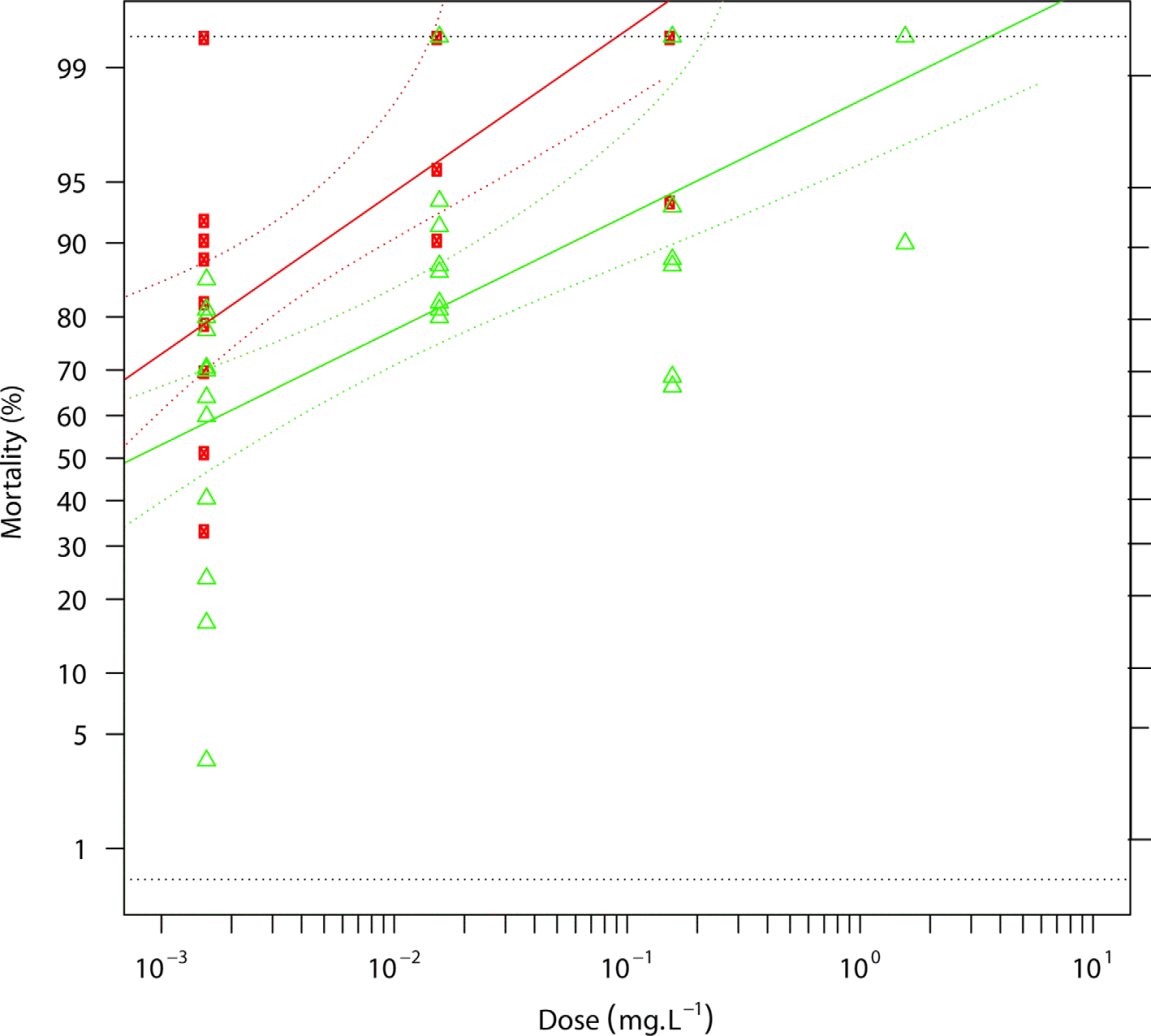
Regression line showing the mortality rates of the Kisumu and M’Bé strains to relative doses of chlorfenapyr. Red squares: Kisumu; green triangles: M’Bé.

Figures 1 and 2 illustrate the resistance status of the *Anopheles gambiae s.s.* population from M’Bé against alpha-cypermethrin (Fig. 1) and chlorfenapyr (Fig. 2). The LC_50_ of alpha-cypermethrin was 24.25 mg/L (95% CI: 19.1–29.8 mg/L) for the population from the M’Bé strain, compared to 0.054 mg/L (95% CI: 0.012–0.144 mg/L) for the susceptible Kisumu strain, corresponding to a high resistance ratio of 450 (Table 1). With chlorfenapyr, the LC_50_ was 0.0007 mg/L (95% CI: 0.0001–0.001 mg/L) for the population from the M’Bé strain, whereas the LC_50_ of the susceptible Kisumu strain was 0.0002 mg/L (95% CI: 0.0001–0.0006 mg/L), indicating a resistance ratio of 3 (Table 1).

**Table 1.** Resistance status of *Anopheles gambiae s.l*. from M’Bé to the two insecticides present in the Interceptor^®^ G2 LN (i.e. alpha-cypermethrin and chlorfenapyr).

Insecticide	Strain	Slope (*SE*)	LC_50_ mg/L (95% CI)	LC_95_ mg/L (95% CI)	Resistance ratio at the LC_50_ (95% CI)
	Kisumu	1.209 (0.2516)	0.054 (0.012–0.144)	1.235 (0.4195–10.348)	–
Alphacypermethrin	M’Bé	1.592 (0.1183)	24.258 (19.163–29.882)	261.762 (195.608–377.923)	450.2 (278.8–726.7)
	Kisumu	0.965 (0.220)	0.0002 (0.0001–0.0006)	0.011 (0.006–0.043)	–
Chlorfenapyr	M’Bé	0.683 (0.126)	0.0007 (0.0001–0.001)	0.195 (0.067–1.510)	3.4 (2.6–4.4)

LC_50_ = the dose required to kill 50% of the mosquitoes exposed to the insecticide, LC_95_ = the dose required to kill 95% of the mosquitoes exposed to the insecticide

### Bio-efficacy of the treatment (WHO cone test)

Table 2 shows the bio-efficacy of each treatment before washing, after washing, and after the field trial in terms of the KD effect and mortality after 3 min and 30 min of exposure.

**Table 2.** The knockdown (KD) rate at 60 min and the mortality rate of Kisumu and the wild resistant strain M’Bé after a 3 min and a 30 min exposure to treated nets following WHO standard procedures (WHO 2013) before washing, after washing, and after the field trial.

		Kisumu						M’Bé					
		3 min exposure			30 min exposure			3 min exposure			30 min exposure		

Times	Treatment	*N*	% KD (60 min)	% Mortality (72 h)	*N*	% KD (60 min)	% Mortality (72 h)	*N*	% KD (60 min)	% Mortality (72 h)	*N*	% KD (60 min)	% Mortality (72 h)
Before washing	Untreated net	212	0^a^	4^a,1^	269	0^a^	3^a,1^	150	0^a^	4^a,1^	155	0^a^	4^a,1^
	Chlorfenapyr-CTN	206	2^a^	18^b,1^	200	16^b^	52^b,2^	99	0^a^	12^b,1^	100	0^a^	24^b,1^
	Interceptor^®^ LN	171	97^c^	99^d,1^	172	100^c^	100^c,1^	172	4^a^	9^ab,2^	55	15^b^	87^c,1^
	Interceptor^®^ G2 LN	167	61^b^	42^c,1^	172	100^c^	81^c,2^	51	0^a^	26^c,1^	180	1^a^	65^b,2^
After washing and before the trial	Untreated net	108	0^a^	3^a,1^	53	0^b^	5^a,1^	51	0^a^	4^a,1^	51	0^a^	4^a,1^
	Interceptor^®^ LN	167	100^b^	100^b,1^	168	100^b^	100^b,1^	52	21^b^	14^a,2^	52	62^c^	17^b,2^
	Interceptor^®^ G2 LN	164	100^b^	100^b,1^	167	100^b^	100^b,1^	108	5^a^	6^a,3^	100	15^b^	24^b,2^
After the trial	Untreated net	158	0^a^	3^a,1^	158	0^a^	3^a,1^	309	0^a^	2^a,1^	309	0^a^	2^a,1^
	Chlorfenapyr-CTN	153	21^a^	56^c,1^	161	39^b^	66^b,1^	103	7^b^	14^bc,2^	112	4^b^	45^c,1^
	Interceptor^®^ LN unwashed	55	93^b^	100^d,1^	56	100^c^	100^d,1^	100	6^b^	27^c,2^	108	4^b^	45^c,2^
	Interceptor^®^ LN 20 washes	51	94^b^	100^d,1^	48	100^c^	100^d,1^	107	23^c^	15^bc,3^	107	28^c^	32^bc,2^
	Interceptor^®^ G2 LN unwashed	53	84^b^	35^b,2^	57	100^c^	86^c,1^	102	0^a^	6^b,3^	109	0^a^	18^b,2^
	Interceptor^®^ G2 LN 20 washes	53	100^b^	100^d,1^	53	100^c^	100^d,1^	98	4^b^	6^b,3^	111	15^c^	20^b,2^

For each strain, values in the same row sharing the same superscript letter do not differ significantly (95% confidence interval). Values in the same line sharing the same superscript number do not differ significantly (95% confidence interval)

Before washing, Interceptor^®^ LN had higher KD (97%) and mortality rates (99%) than Interceptor^®^ G2 (61% KD, 42% mortality) (*p* < 0.05) for the susceptible Kisumu strain after a 3 min exposure, whereas after a 30 min exposure, both Interceptor^®^ and Interceptor^®^ G2 induced 100% KD and 100% and 81% mortality, respectively.

After washing and before the field trial, both Interceptor^®^ and Interceptor^®^ G2 induced a 100% KD effect and mortality after 3 min and 30 min of exposure.

After the field trial, Interceptor^®^ unwashed and washed 20 times and Interceptor^®^ G2 washed 20 times induced significantly higher mortality (100%) than unwashed Interceptor^®^ G2 (35%) (*p* < 0.05) after a 3 min exposure. However, after a 30 min exposure, the unwashed Interceptor^®^ G2 induced 86% mortality.

Before the trial, Chlorfenapyr-CTN did not induce high mortality (ranging from 2% and 39%), irrespective of the exposure time. After the trial, chlorfenapyr-CTN induced 56% and 66% mortality after a 3 min and a 30 min exposure, respectively.

In terms of the resistant M’Bé strain, it is interesting to note that a 30 min exposure to unwashed Interceptor^®^ and Interceptor^®^ G2 induced 87% and 65% mortality, respectively, whereas these LLINs when washed 20 times induced 17% and 24% mortality, respectively (*p* < 0.05). After the trial, this difference based on the extent of being washed was no longer significant. Moreover, it is noteworthy that chlorfenapyr-CTN induced a higher mortality than Interceptor^®^ G2 against the population from M’Bé after a 30 min exposure.

When we compared the resistant population from M’Bé to the Kisumu strain, we observed that washing Interceptor^®^ and Interceptor^®^ G2 20 times induced very low mortalities (≤ 15%) versus 100% for the Kisumu strain after a 3 min exposure time.

### Experimental hut trial

Table 3 shows the efficacy of all of the treatments in terms of deterrence, induced exophily, blood-feeding inhibition, and induced mortality.

**Table 3.** Summary results of the experimental hut trial against wild free-flying resistant *Anopheles gambiae s.s.* mosquito from M’Bé.

	Control	Chlorfenapyr-CTN	Interceptor^®^ LN		Interceptor^®^ G2	
			Unwashed	Washed 20 times	Unwashed	Washed 20 times
Total collected	611	314	255	348	305	369
Average caught/night	17^a^	9^b^	7^b^	9^b^	8^b^	10^b^
Deterrence (%)	–	49	58	43	50	40
% caught in net (CI)	42 (35–48)^a^	8 (4–11)^d^	20 (14.9–26)^b^	33 (26–39)^c^	15 (10–20)^b^	21 (16–27)^b^
Total females in veranda trap	180	180	114	160	204	202
Exiting % (CI)	29 (24–33)^a^	59 (51–66)^c^	45 (37–53)^b^	44 (37–51)^b^	64 (57–71)^c^	51 (43–58)^b^
Induced exiting (%)	–	40	23	22	53	36
Total females blood-fed	300	71	128	158	86	120
Blood-feeding % (CI)	56 (49–63)^a^	25 (19–31)^d^	54 (46–62)^a^	49 (41–56)^ab^	36 (28–43)^bc^	41 (33–48)^c^
Blood-feeding inhibition %	–	54	NS	NS	43	34
Personal protection %	–	76^a^	57^bc^	47^c^	71^ab^	60^b^
72 h mortality % corrected based on the control	–	92 (89–95)^a^	10 (6–13)^c^	11 (7–14)^c^	87 (85–93)^ab^	82 (77–85)^b^

Values along each line sharing the same superscript letter do not differ significantly (95% confidence interval).

#### Deterrence

A total of 2202 *Anopheles gambiae s.l.* mosquitoes were collected over the 36 nights of the trial. In the control hut, 611 *An. gambiae* were caught (i.e. a mean number of 17 per night). All of the treatments resulted in significant deterrence compared to the untreated net (*p* < 0.001).

#### Exophily

All of the treated nets induced higher exiting of *An. gambiae s.l.* than the untreated control nets (29%; *p* < 0.001). Unwashed Interceptor^®^ G2 had higher exit rates (53%) compared to Interceptor^®^ (*p* < 0.001). The induced exophily by Interceptor^®^ G2 that was washed 20 times (36%), unwashed Interceptor^®^ (23%), and Interceptor^®^ that was washed 20 times (22%) were not significantly different (*p* > 0.05).

#### Blood-feeding rate and personal protection

Blood-feeding rates were significantly different in all treatment arms relative to untreated nets (56%) (*p* = 0.001), except for Interceptor^®^ that was unwashed (54%) or washed 20 times (49%) (*p* > 0.05) (Table 3). The blood-feeding inhibition induced by Interceptor^®^ G2 that was unwashed or washed 20 times and chlorfenapyr-CTN was 43%, 34%, and 54%, respectively (Table 3). Whereas all of the treatment arms conferred a high level of personal protection against the wild resistant population of *An gambiae s.l.*, there was no difference in the level of personal protection between Interceptor^®^ G2, Interceptor^®^, and chlorfenapyr-CTN (*p* = 0.379). Interceptor^®^ G2 washed 20 times provided more personal protection (60%) than Interceptor^®^ LN washed 20 times (47%) (*p* = 0.01).

#### Mortality rate

**Figure 3.**
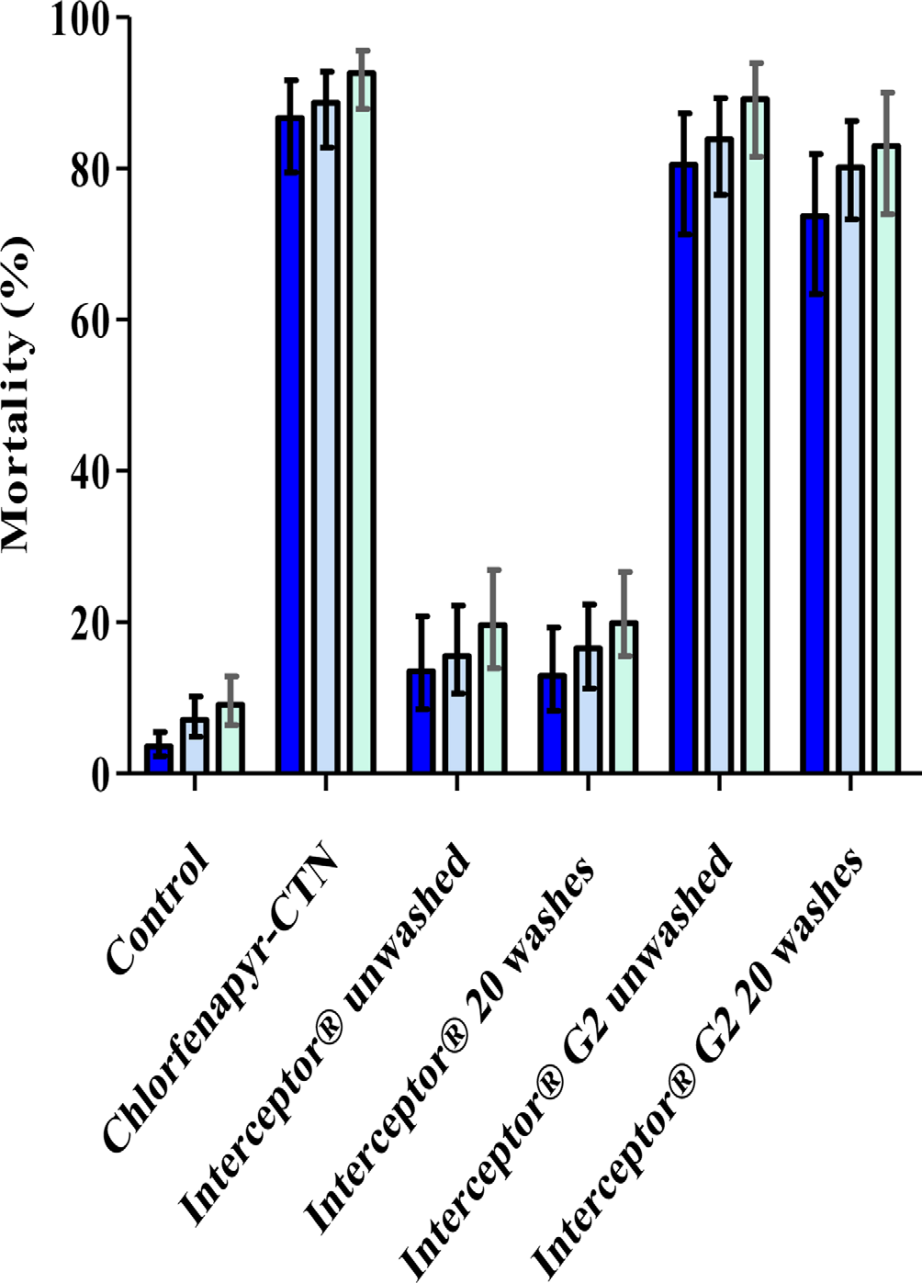
Mortality of *An. gambiae s.l.* during overnight, 24 h, and 72 h after exposure to different nets in the experimental huts. Deep blue: immediate mortality; intermediate blue: mortality after 24 h; light blue: mortality after 72 h.

The mortality rate with the untreated net was 9%. The corrected mortality rates recorded with Interceptor^®^ unwashed and washed 20 times after 72 h of holding were 10% and 11%, respectively (Table 3). There was no difference between the lethal effect of these three treatment arms (*p* = 0.28). Interestingly, the mortality induced by Interceptor^®^ G2 treated with the mixture of alphacypermethrin-chlorfenapyr was significantly higher compared to Interceptor^®^ treated with alphacypermethrin alone (*p* < 0.001). The mortality induced by Interceptor^®^ G2 washed 20 times (82%) did not differ from unwashed Interceptor^®^ G2 (87%) (*p* = 0.16). Chlorfenapyr CTN induced higher mortality (92%) than the other treatments (*p* < 0.05), except Interceptor^®^ G2 unwashed, which induced a similar mortality rate (*p* > 0.05). Furthermore Interceptor^®^ G2 and chlorfenapyr-CTN immediately killed 78% to 88% of all mosquitoes (Fig. 3).

## Discussion

Pyrethroid resistance is now widespread in Sub-Saharan Africa [39]. Novel insecticides to manage pyrethroid resistance are critical to maintain the efficacy of malaria vector control. The pyrrole insecticide chlorfenapyr has been identified as a novel insecticide of interest to public health. Its combination with alpha-cypermethrin on bed nets has yielded promising results [27, 31]. The purpose of this study was to assess the efficacy of the new long-lasting mixture net, Interceptor^®^ G2, against wild pyrethroid-resistant *An. gambiae s.l.* in a semi-field trial in Côte d’Ivoire. This new LLIN was compared to standard Interceptor^®^ impregnated with the same pyrethroid and nets hand-treated with chlorfenapyr to determine whether the mixture of alpha-cypermethrin and chlorfenapyr on this new LLIN is effective at controlling resistant malaria vectors. The phase II trial was carried out in an area where *An. gambiae s.l.* is multi-resistant to pyrethroids, carbamates, and organochlorides [7, 17, 48]. In the present trial, Interceptor^®^ impregnated with alpha-cypermethrin induced low mortality rates (around 10%). Moreover Interceptor^®^ did not inhibit blood-feeding. This relatively low level of personal protection relies only on the deterrent effect of Interceptor^®^. The contrast with results reported for a study in Tanzania is striking, where the same Interceptor^®^ (unwashed and washed 20 times) induced 92% and 76% mortality, respectively, in experimental huts against susceptible *An. gambiae s s.* [20]. This pronounced difference highlights the phenotypic impact of insecticide resistance in the M’Bé area, with a confirmed LC_50_ resistance ratio of 450.15. This pronounced difference is reason for grave concern because such resistance levels are widespread in Côte d’Ivoire [7] and in the entire West-African region [9, 38]. The new Interceptor^®^ G2 (unwashed or washed 20 times) and chlorfenapyr-CTN induced far more blood-feeding inhibition (>34%) and mortality (>80%) than standard Interceptor^®^ against this multi-resistant *An gambiae s.l.* population of M’Bé. In the present study, the mortalities scored immediately, 24 h and 72 h after collection were not significantly different. These results support the hypothesis that scoring the mortality at 24 h is sufficient to measure the chlorfenapyr-induced mortality, even though this insecticide acts more slowly than pyrethroids [23]. Nevertheless, there are some contrasting reports in the literature [30]. In the hut trials, Interceptor^®^ G2 and chlorfenapyr nets killed more free-flying mosquitoes (three-fold) relative to standard Interceptor^®.^ This outcome is similar to that found by N’Guessan *et al.* [25] in Benin and Bayili *et al.* [4] in Burkina Faso. This high mortality of resistant malaria vectors is promising for overcoming the threat of resistance in such malaria vector populations. WHOPES sets criteria for testing and it recommends LLIN for malaria programs. In experimental huts that simulate domestic conditions and that provide a definitive test of LLIN efficacy [23], Interceptor^®^ G2 outperformed the WHOPES recommended Interceptor and hence meets the WHO criteria for interim recommendation and for undergoing a phase III study at the community level.

Interestingly, unwashed candidate LLINs that should reach efficacy thresholds with cone tests [43] (before washes, after washes and after the trial) did not do so before the washes and after the trial. Indeed, in terms of efficacy against the Kisumu strain, both unwashed and washed Interceptor^®^ and Interceptor^®^ G2 washed 20 times met the WHOPES criteria (95% KD or 80% mortality) after a 3 min exposure in WHO cone tests, whereas a 30 min exposure was required for unwashed Interceptor^®^ G2 LN to reach these efficacy thresholds [1, 14]. These results raise two questions: 1) what is the impact of the washing procedure on the bio-availability of the active compounds? and 2) Is the 3 min exposure relevant to assess the bio-efficacy of a new generation LLINs in the laboratory?

Our results support the hypothesis that the washing procedure increased the bio-availability of the active ingredient on the net surface [14, 16]. In this context, it would be interesting to focus on bio-availability dynamics in laboratory studies available in the literature.

The WHO standard cone bioassay exposure time to assess bio-efficacy of LLINs in the laboratory is three minutes. However, it should be emphasized that the time mosquitoes spend in contact with the net is influenced by the contact-irritancy of the insecticide [22]. One can imagine that mosquitoes that are highly irritated do not spend enough time on the treated net during the bioassay to render the results relevant in terms of both KD and mortality. Authors such as Oxborough *et al*. [32] have therefore stated that some classes of insecticide such as chlorfenapyr may require longer exposures to induce mortality that is closer to the mortality of free-flying mosquitoes.

For the multiresistant M’Bé population [7, 17], all of the bed nets induced lower KD and mortality rates after a 3 min exposure than a 30 min exposure. Nevertheless, mortality rates – even after a 30 min exposure – did not exceed 45%, whereas chlorfenapyr-CTN, Interceptor^®^ G2 unwashed and washed 20 times induced 82%–92% mortality against the wild population from M’Bé in experimental huts. By contrast, Interceptor^®^ unwashed and washed did not induce more than 11% mortality in the experimental huts, whereas they induced 45% and 32% mortality, respectively, after 30 min exposure tests. These contradictory results once again highlight the difficulty with extrapolating results from the laboratory setting.

In contrast to previous studies on mixture-impregnated nets [33–35], we were not able to detect any positive interaction (i.e. a synergistic effect) in terms of induced mortality due to the chlorfenapyr-CTN induced mortality rate of 92%. Chlorfenapyr-CTN also displayed the highest level of blood-feeding inhibition. This interesting result raises an obvious question: would it be relevant to develop a long-lasting net impregnated with chlorfenapyr alone? It is likely that the cost-effectiveness would be increased and that the absence of alphacypermethrin would contribute to the pyrethroid-resistance management plan.

Chlorfenapyr is a non-neurotoxic slow-acting insecticide that exerts its effect by disrupting metabolic respiratory pathways in the mitochondria of cells [6]. Balmert *et al*. [3] have shown that the expression of cytochrome P450 s involved in oxidative metabolism are under circadian control and expressed more at night during anopheline flight and host-seeking activity. This would explain the high level of mortality in experimental huts against active mosquitoes and it would also account for the low mortality in day-time bioassays. However, this study revealed reduced susceptibility to the chlorfenapyr technical ingredient (a resistance ratio of 3 at the LC_50_), raising the question of metabolic mechanisms that might induce cross-resistance in the M’Bé area. Nevertheless, chlorfenapyr remains a more appropriate insecticide due to its effectiveness to kill pyrethroid-resistant Anopheline mosquitoes. However, it would also be interesting to monitor changes in susceptibility to this insecticide and all new active ingredients that might be added to the vector control arsenal. The fight against malaria transmission must involve integrated vector management relying on several efficient tools.

## Conclusion

Interceptor^®^ G2 met the WHOPES criteria to undergo a phase III study at the community level. The entomological data are very encouraging, indicating that investigation of its efficacy in Phase III is warranted. It would be desirable to undertake randomized controlled trials dealing with epidemiological outputs to study the potential of Interceptor^®^ G2 LN to better protect communities in areas where there is insecticide resistance. It is undeniable that this new LLIN which now has an interim WHO/PQ recommendation will expand the arsenal to fight resistant malaria vectors, although the scientific community must monitor susceptibility to this new active ingredient in parallel with the potential implementation of this new LN.

## Conflict of interest

The study, which took place in the framework on the Anopheles, Biology and Control (ABC) network, was supported by the World Health Organization (WHO), which approved the decision to publish the findings. The authors declare that they have no competing interests.
